# Detecting Neuroimaging Biomarkers for Psychiatric Disorders: Sample Size Matters

**DOI:** 10.3389/fpsyt.2016.00050

**Published:** 2016-03-31

**Authors:** Hugo G. Schnack, René S. Kahn

**Affiliations:** ^1^Department of Psychiatry, Brain Center Rudolf Magnus, University Medical Center Utrecht, Utrecht, Netherlands

**Keywords:** machine learning, effect size, heterogeneity, classification and prediction, neuroimaging, schizophrenia

## Abstract

In a recent review, it was suggested that much larger cohorts are needed to prove the diagnostic value of neuroimaging biomarkers in psychiatry. While *within* a sample, an increase of diagnostic accuracy of schizophrenia (SZ) with number of subjects (*N*) has been shown, the relationship between *N* and accuracy is completely different *between* studies. Using data from a recent meta-analysis of machine learning (ML) in imaging SZ, we found that while low-*N* studies can reach 90% and higher accuracy, above *N*/2 = 50 the maximum accuracy achieved steadily drops to below 70% for *N*/2 > 150. We investigate the role *N* plays in the wide variability in accuracy results in SZ studies (63–97%). We hypothesize that the underlying cause of the decrease in accuracy with increasing *N* is sample heterogeneity. While smaller studies more easily include a homogeneous group of subjects (strict inclusion criteria are easily met; subjects live close to study site), larger studies inevitably need to relax the criteria/recruit from large geographic areas. A SZ prediction model based on a heterogeneous group of patients with presumably a heterogeneous pattern of structural or functional brain changes will not be able to capture the whole variety of changes, thus being limited to patterns shared by most patients. In addition to heterogeneity (sample size), we investigate other factors influencing accuracy and introduce a ML effect size. We derive a simple model of how the different factors, such as sample heterogeneity and study setup determine this ML effect size, and explain the variation in prediction accuracies found from the literature, both in cross-validation and independent sample testing. From this, we argue that smaller-*N* studies may reach high prediction accuracy at the cost of lower generalizability to other samples. Higher-*N* studies, on the other hand, will have more generalization power, but at the cost of lower accuracy. In conclusion, when comparing results from different ML studies, the sample sizes should be taken into account. To assess the generalizability of the models, validation (by direct application) of the prediction models should be tested in independent samples. The prediction of more complex measures such as outcome, which are expected to have an underlying pattern of more subtle brain abnormalities (lower effect size), will require large samples.

## Introduction

Since the recent development and application of machine learning (ML) techniques in neuroimaging data for classification and prediction of psychiatric disorders, dozens of studies have been published on the use of (structural and functional) MRI scans for, e.g., classification of schizophrenia (SZ) [for a recent overview, see Ref. (1)], autism (2), ADHD (3), the separation of bipolar disorder from SZ (4), prediction of outcome/illness course in SZ and related psychotic disorders (5, 6), transition to psychosis (7), and distinguishing prodromal from first-episode psychosis (8) at the level of the individual. The published values of the prediction accuracy, the standard measure of the performance, of the binary classification models, range from a little above 50% (rolling the dice) up to (and including!) 100% (1, 9, 10). The sample sizes used in these studies vary between 15 and 198 per diagnostic group. Although, such as in group-level statistics, it has been shown that sample size matters and that classification accuracy in small samples show large variation (11), a substantial number of studies with low *N* has been published. While this is usually justified for pilot studies, proof of principle studies, and studies on “difficult” samples (difficult inclusion or using challenging imaging techniques), they do not support conclusions about the potential of the technique to be used in a clinical setting. It has recently been suggested that ML studies with larger samples should be conducted in order to be of diagnostic value (12) and we think the time has come to make the step toward a next “generation” of ML studies, using large samples and/or independent validation samples and (re)use (smaller) studies. In this work, we will try to interpret the variations in performance seen in published ML neuroimaging studies. To do this, we will introduce the ML effect size as a measure of the predictive power of a model and develop a theoretical model to quantify the relationship between sample heterogeneity and prediction accuracy. We will conclude with a number of recommendations for psychiatric neuroimaging ML studies in the future.

### Machine Learning Studies in Psychiatric Imaging: Schizophrenia

**Figure 1:**
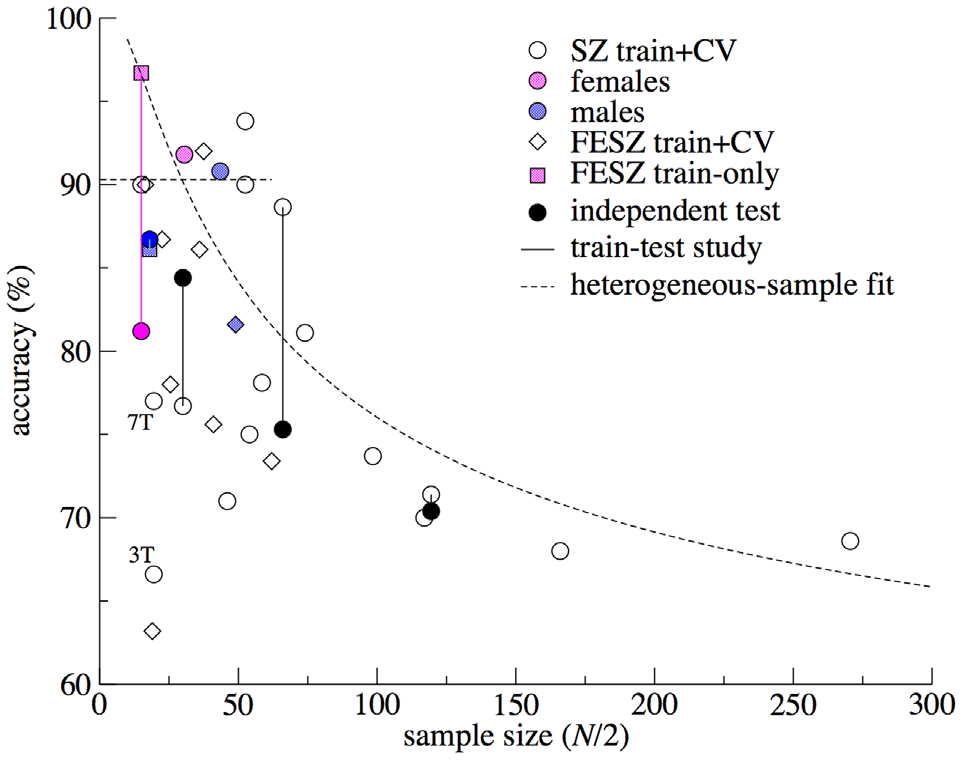
**Prediction accuracy versus sample size for the schizophrenia machine learning studies using structural MRI**. Data are taken from the reviews by Zarogianni et al. (9) and Kambeitz et al. (1) and some (recent) studies (4, 14–18). Sample size *N*/2 was calculated as the mean of the patient and control sample sizes. Different symbols mark cross-validation accuracy from studies on chronic/mixed patients (circles) and first-episode patients (diamonds). Soft colors were used to indicate studies that included only females (pink) or males (blue). Train accuracy without cross-validation is marked by squares. Lines connect train–test studies, where the accuracy in the independent test sample is marked with solidly filled symbols. “3T” and “7T” mark the study by Iwabuchi (19), using the same subjects scanned at different field strengths. Curved dashed line: heterogeneous-sample theory. Horizontal dashed line: stretched range of homogeneous samples.

We base our approach on the observations from published sMRI-ML studies on SZ summarized in Figure 1. The figure clearly illustrates two phenomena: (1) published ML models from smaller samples yield higher classification accuracies and the observed accuracies appear to lie on (and below) a line that divides the diagram in two and (2) replications in independent validation samples yield lower accuracies, also decreasing with (training) sample size. These effects may be explained by (at least) two reasons: sample homogeneity and publication bias. Poorer performing models in small samples may remain unpublished, while this variation in accuracy may be due to chance (11) as well as better performance in homogeneous (small) samples. The lower accuracy in the larger samples can be ascribed to increased within-sample heterogeneity; these models may better capture the “complete picture” of SZ patterns. These models generalize better to other samples drawn from different populations at the cost of a lower accuracy. From all applications of ML in neuroimaging, those in psychiatry seem to be affected most severely by the effects of small samples, given the heterogeneous nature of the disorders, both in appearance and in underlying brain features (13). In the following, we will setup a simple theoretical framework to describe the different factors that affect a prediction model’s performance. The resulting formulas can be used to (1) quantitatively relate the observed fall in classification accuracy for increasing sample size to *within-sample* heterogeneity and (2) determine the *between-sample* heterogeneity, i.e., the non-overlap in sample characteristics from the accuracy difference in a test/retest study. These tools can also be applied to (*post hoc*) multicenter ML studies to unravel the heterogeneity of the brain biomarker structure related to psychiatric disorders.

## Methods and Results

### Accuracy of Machine Learning Models: Effect Size

**Figure 2:**
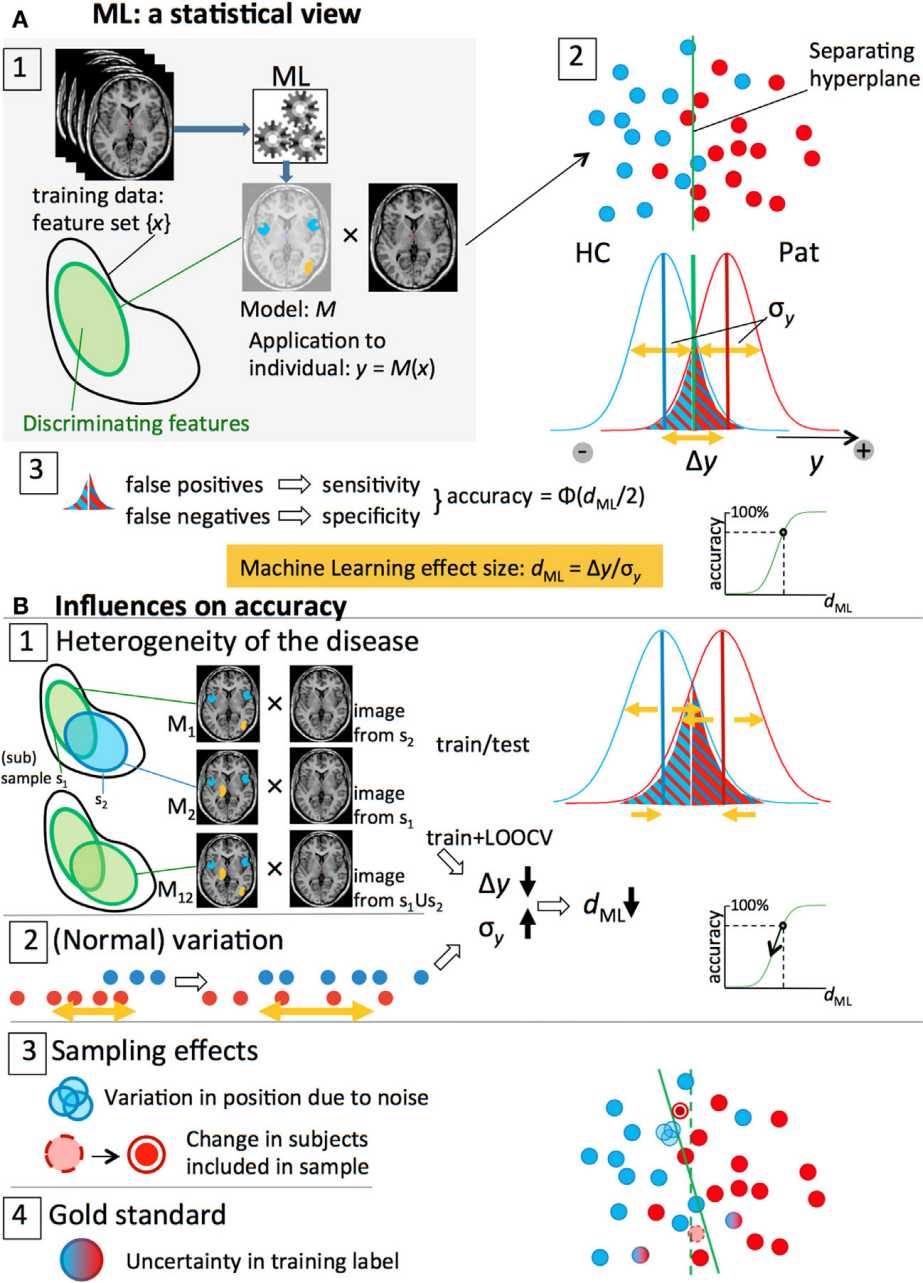
**Overview of the statistical side of machine learning in neuroimaging data and the different factors that influence prediction accuracy**. **(A)** 1. An ML algorithm is trained on a set of labeled, preprocessed, MRI scans, resulting in a model M that classifies patients and controls based on a discriminative subset of the features (feature vector). 2. The classification is done by an (optimal) separating hyperplane in the (high-dimensional) feature space. Application of the model to an individual scan yields an output value *y* that is proportional to the distance of the subject’s feature vector to the plane: blue (HC) and red (Pat) dots. The *y*-values of all subjects form two distributions with widths σ*_y_* and means separated by a distance Δ*y*. 3. A threshold halfway the distributions separates the two groups; the overlapping parts below and above the threshold represent the false negatives and false positive, respectively. For symmetrical distributions, the accuracy can be estimated from the ML effect size, *d*_ML_ = Δ*y*/σ*_y_*. **(B)** 1, 2. Heterogeneity of the disease and (normal) variation in brain measures lead to lower Δ*y* and larger σ*_y_* and, thus, smaller effect sizes and classification accuracies. 3, 4. Sampling effects and noise and imperfect expert labeling cause uncertainties in the positions of the subjects and affect the separating hyperplane and (test) accuracy.

**Figure 3:**
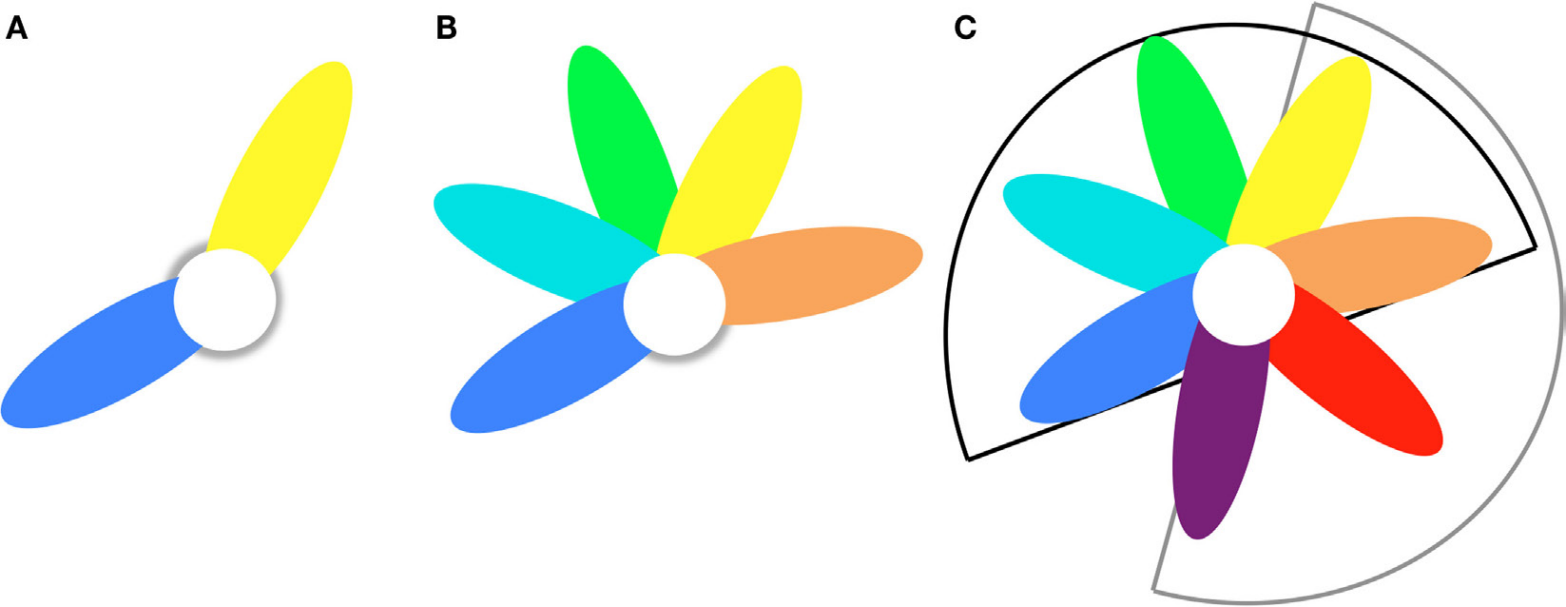
**Petal model to describe disease heterogeneity within and between samples**. **(A)** Two-fold heterogeneity sample consists of blue and yellow subsamples that have shared (white) and unique (colored) brain abnormalities. **(B)** *H*-fold heterogeneity sample consists of *H* = 5 subsamples that have shared (white) and unique (colored) brain abnormalities. **(C)** Train/test heterogeneity The model trained on sample B (*H* = 5; black sector) is tested on a sample with *H′*-fold heterogeneity (*H′* = 4; grey sector). The overlap is *T*-fold (*T* = 2; yellow and orange petals).

Figure 2A summarizes the process of applying ML to imaging data and the resulting classification performance. Every subject is represented by a so-called feature vector *x* that contains the features, or measures, that will be used to separate the two groups. These features can consist of any set of (neuroimaging) measures, for instance, a set of atlas-based regional brain volumes (low-dimensional feature space), or voxelwise gray matter densities (high-dimensional feature space) (Figure 2A-1). The features {*x_*i*_*} are distributed around a mean *x* with SD σ*_*x*_*. In group-wise statistics, effect size Cohen’s *d* is defined as *d* = (*x*_1_ − *x*_0_)/σ*_*x*_*, which is a measure indicating how large the group-effect in this feature is in relation to the variation observed in this feature between subjects. The significance of this difference can be expressed by *t* = *d*√*N* that can be converted to a *p*-value. From this formula, it is clear that, even for very small effect sizes *d*, a group difference can become “significant” by increasing *N*. This, however, has of course no effect on the overlap between the distributions. Suppose we would use this feature to separate the individuals of the two groups, we would draw a line as indicated in Figure 2A-2: the overlap of the distributions shaded in the Figure indicates the wrongly classified individuals. The non-overlap reflects the fraction of individuals that is correctly classified [Cohen’s non-overlap measure U_2_, see Ref. (20)]. Under the assumption that the distributions of the feature are normal and equal for the two groups, this fraction may be estimated as Φ(*d*/2), where Φ(.) is the cumulative normal distribution (Figure 2A-3). While effect sizes of 0.8 and larger are commonly referred to as “large” (20) and would give rise to a “very significant” group difference *t* = 8 for *N* = 100, only 66% of the individuals would be assigned to the correct class in this case. This shows the fundamental difference between parameters that are significantly different between groups and their use for making individual predictions. This is one of the reasons that univariate prediction models are rare and that multivariate techniques are invoked to make use of the combined predictive power of many variables.

#### Machine Learning Effect Size

In the following, we will expand the univariate effect size to an effect size related to the output of multivariate ML models. Under application of a prediction model, a subject’s feature set {*x_*i*_*} is transformed into a single value *y*, representing the outcome of the classifier (Figure 2A-1). For a binary classifier, values of *y* > 0 indicate a subject is classified as belonging to group “+1” (for instance, the patient group), and values of y < 0 indicate group “−1” membership (for instance, the control group). The distribution of *x* ± σ*_*x*_* is transformed accordingly, yielding *y* ± σ*_*y*_* (Figure 2A-2). In analogy to the group-level effect size, a ML effect size can be defined as *d*_ML_ = Δ*y*/σ*_*y*_*. Here, Δ*y* = *y*_1_ − *y*_0_ is the “separation strength,” i.e., the distance between the mean classification output values of the two groups, *y*_0_ and *y*_1_, respectively; the spread of the classifier’s output around the group means is represented by σ*_*y*_*, estimated as the pooled SD, √(12sy02+12sy12). The larger σ*_*y*_* with respect to Δ*y*, the more subjects will be wrongly classified (Figure 2A-3). The classification accuracy, defined as the fraction of correctly classified subjects, may, again, be estimated as Φ(*d*_ML_/2) (Figure 2A-3). As we saw in the previous paragraph, “large” effect sizes (>0.8) will produce only moderate prediction accuracies (66% for *d*_ML_ = 0.8), which, from a diagnostic point of view, is not considered as being “large.” A new scale should be defined for interpretation of ML effect sizes. For binary classification models, we suggest to label accuracies <60% (*d* = 0.50) as small; 60–70% (*d* = 1.05) as “modest”; 70–80% (*d* = 1.68) as “medium”; and >80% as “large” (see Table 1). In the following, we will identify the sources that influence prediction accuracy (via *d*_ML_) that play a role in (neuroimaging) ML studies. This will help understanding the meaning of a certain published model together with its prediction accuracy.

**Table 1:** **Effect sizes, statistical and machine learning**.

Effect size	Cohen’s qualification	*t*/*p*		Machine learning	
		*N* = 50	*N* = 200	Accuracy (U_2_, %)	Proposed qualification
0	–	0	0	50	
0.4	“Small”	1.41/0.16	2.83/0.005	58	“Small”
0.6	“Medium”	2.12/0.04	4.24/3.10^−5^	62	“Modest”
0.8	“Large”	2.83/0.01	5.66/<10^−5^	66	“Modest”
1.05–1.68	–	3.71/5.10^-4^	7.42/<10^−5^	70–80	“Medium”
>1.68	–	5.94/<10^-5^	11.9/<10^−5^	>80	“Large”

**N* is the total sample size; calculations assuming normally distributed data*.

### Accuracy of ML Models: Gold Standard, Training, Testing, and Heterogeneous Samples

**Figure 4:**
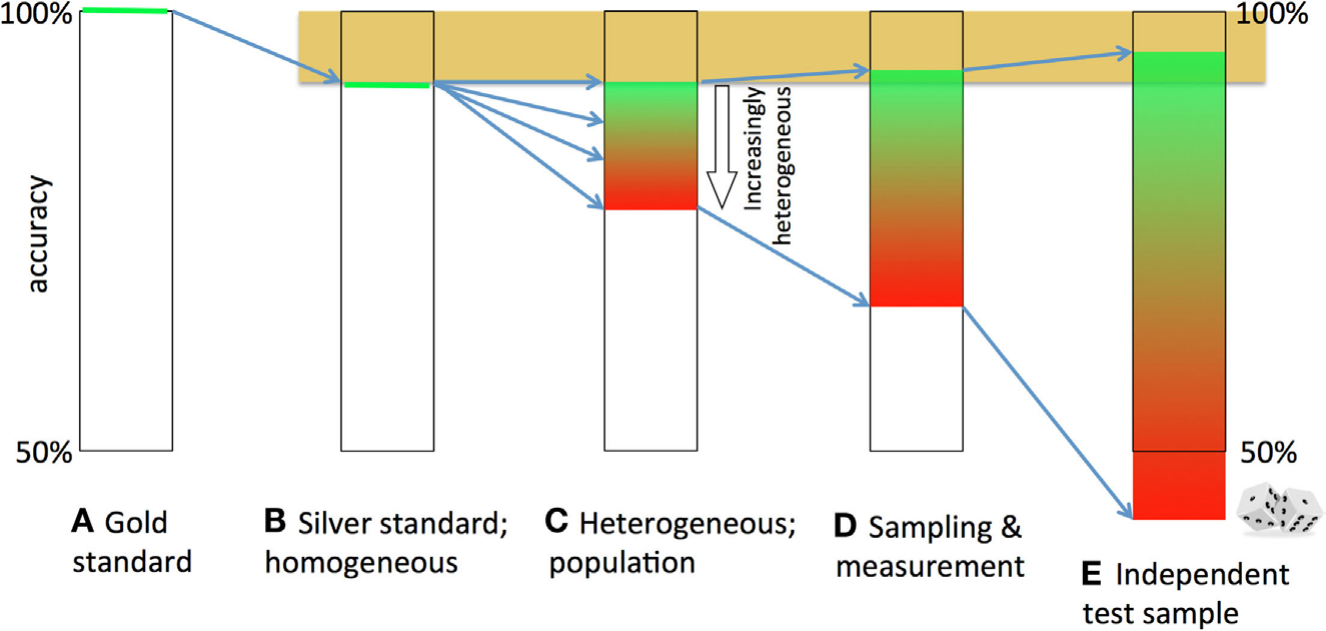
**Decay and broadening of the prediction accuracy**. The theoretically attainable 100% accuracy **(A)** is lowered, because the gold standard is imperfect **(B)**. If the brain abnormalities underlying the disease are heterogeneous, no straightforward (linear) model can classify all patients correctly **(C)**. Sampling effects, biological variation and measurement noise further lower and broaden the accuracy **(D)**. Finally, if a model is tested in an independent sample, the accuracy becomes lower due to between-sample heterogeneity and broader due to sampling effects **(E)**.

We will show that there are basically four “channels” through which the performance of a classifier is influenced (subsections 1–4; Figures 2B-1–4; Table 2). Depending on the kind of study and the way performance is assessed, different sources play a role. We present the ideas and some resulting formulas and numerical results for simple cases here. The derivation of the formulas is given in the Datasheet S1 in Supplementary Material. The results are then related to the observations made from Figure 1. To investigate the influence of the different sources, we will consider two types of ML prediction studies: (i) the single-sample study, where a model is trained and tested using (*k*-fold; leave-one-out) cross-validation (CV) in the same sample and (ii) the two-sample study where a model trained on one sample (the discovery sample) is tested in an independent second sample (the validation sample).

**Table 2:** **Factors that influence the ML effect size and classification accuracy**.

Source	Relevant factor	Acting on	Effect in study:	
			Training + LOOCV	Train → test
Reference: homogeneous sample (“0”)	Separation strength, Δ*y*_0_ Spread, σ*_y_*_0_	–	*d*_ML0_ = Δ*y*_0_/σ*_y_*_0_ *acc*_0_ = Φ(*d*_ML0_/2)	–
Heterogeneity of the disease^a^	*f*	Separation strength, Δ*y* → *d*_ML_	*d*_ML_ = √[(1 + *f*)/2] *d*_ML0_	*d*_ML_ = *fd*_ML0_
Heterogeneity: biological variation	σ_BIOL_	broadening, σ*_y_* → *d*_ML_	*d*_ML_ = (σ*_y_*_0_/σ*_y_*)*d*_ML0_	*d*_MLT_ = (σ*_y_*/σ*_y_*_T_)*d*_ML_
Measurement noise	σ_EXP_	broadening, σ*_y_* → *d*_ML_	*d*_ML_ = (σ*_y_*_0_/σ*_y_*) *d*_ML0_	*d*_MLT_ = (σ*_y_*/σ*_y_*_T_)*d*_ML_
Sampling effects (finite *N*)	*N*, σ*_y_*	uncertainty in accuracy	≤ SD(*acc*) in train/test case	SD(*acc*) = √(*acc*_true_ × (100%−*acc*_true_)/*N*)
Gold → silver standard	Intra-class kappa, κ	ceiling of accuracy		*acc* = κ × *acc*_0_ +(1−κ)/2

*^a^i.e., related to the prediction model*.

**f* = cos(θ), the relative amount of heterogeneity;σ^2^*_y_* = σ^2^_BIOL_ + σ^2^_EXP_; *acc* = accuracy; subscripts “T” refer to values in the Test sample*.

#### 1. Heterogeneity of the Disease

Heterogeneity of the disease shows up as different parts of the brain being affected by the disease in different patients. This has a direct influence on the set of discriminating features and thus on Δ*y*. Examples of source of disease heterogeneity are differences in: subtypes of the disorder (e.g., by symptoms, see Ref. (21)), disease status of the patients: illness severity (outcome) and course [age-of-onset and illness duration (first-episode patients versus chronic patients) and number of psychoses, etc.]; medication use [type and (cumulative) dose], etc.; differences in genetic background (as far as they influence the disease). However, much of the heterogeneity will not be attributable to clear factors. Disease heterogeneity has an effect in both the training sample (within-sample heterogeneity) and an independent test sample (between-sample heterogeneity).

##### 1a. Testing a Model’s Accuracy in an Independent Validation Sample: Heterogeneity in the Population, Causing between-Sample Heterogeneity

We start with training a disease prediction model in a homogeneous training sample (s_1_, see Figure 2B-1). More precisely, strict inclusion criteria on the demographic and clinical parameters (e.g., age, gender, duration of illness) will lead to a clinically homogeneous sample. In such a sample, the disease-underlying brain abnormalities may be assumed to be as homogeneous as possible too. The prediction model (M_1_) will find a “clear” discriminative pattern in these brain abnormalities and be able to transform the features {*x_*i*_*} into a decision value *y* that can separate the patients from the controls with high accuracy. Suppose we now wish to test the model’s validity in an independent sample (s_2_) that is derived from a different population and/or with different inclusion criteria (e.g., it has a different duration of illness or genetic background of the disease). Sample 2 is, thus, heterogeneous with respect to sample 1. Put differently, part of the (clinical) parameters is the same, but another part is different. The samples are said to be mutually heterogeneous, i.e., exhibit between-sample heterogeneity. We expect that this clinical heterogeneity is reflected by a heterogeneity of the underlying brain patterns too (Figure 2B-1). The model M_1_ will not be sensitive to any brain abnormalities present in s_2_ but not in s_1_, and the part of the model that is based on abnormalities present in s_1_ but not in s_2_ will not contribute to the separation of cases and controls in s_2_. Thus, only the pattern of shared abnormalities (the homogeneous part) will be of use for the classification of sample 2. For a linear prediction model M_1_ and samples s_1_ and s_2_ that share a fraction *f*_12_ of discriminative features we can show that the separation strength in s_1_, Δ*y*, is in s_2_ lowered according to this fraction: *f*_12_Δ*y*. The ML effect size is lowered by the same factor: *d*_ML_ → *f d*_ML_. In the range of 70–90% prediction accuracies for M_1_, the resulting drop in accuracy in s_2_ is approximately given by: Δ*acc* ≈ 53% × ^10^log *f*_12_ (see Datasheet S1 in Supplementary Material). For example, a 50% overlap in discriminative features between the samples (*f*_12_ = 0.5) will result in a drop of 16% classification accuracy in the test sample as compared to that obtained in the training sample.

##### 1b. (Cross-Validation) Accuracy of the Model: Heterogeneity within Larger Training Samples

In Section “Testing a Model’s Accuracy in an Independent Validation Sample: Heterogeneity in the Population, Causing between-Sample Heterogeneity,” we considered the case of homogeneous samples. Such samples will be relatively small in practice. Larger samples will inevitably be heterogeneous because of the difficulty to include a large amount of subjects fulfilling the strict inclusion criteria. In the following, we will assume that this heterogeneous sample is constituted of two or more homogeneous subsamples. Part of the properties is shared between the subsamples, but other properties differ (e.g., duration of illness). For the case of two subsamples, the situation is the same as described in the previous section (Figures 2B-1 and 3A), but now a model M_12_ is trained on the combined s_1_ + s_2_ sample. The model will be a weighted average of the (hypothetical) models from the homogeneous subsamples. The amount of within-sample heterogeneity is determined by both the number of homogeneous subsamples the sample can be divided into and the overlap between the discriminative feature sets (Figures 2B-1 and 3A,B). The larger the within-sample heterogeneity, the lower the separation strength Δ*y* will be (*cf*, see Testing a Model’s Accuracy in an Independent Validation Sample: Heterogeneity in the Population, Causing between-Sample Heterogeneity). Furthermore, different sets of discriminative features will generally also lead to a larger pool of discriminative features that will not contribute to the discrimination in most subjects but will have random effects (affecting σ*_*y*_*). The net effect is a decrease of the effect size *d*_ML_. Training (CV) accuracy will be lower as compared to a (hypothetical) homogeneous sample (of the same size) and depend on the within-sample heterogeneity, which, for a two-subsample case is defined by the fraction of shared features, *f*_12_. The effect size is attenuated as follows: √((1 + *f*_12_)/2). (See section C of the Datasheet S1 in Supplementary Material for the derivation of this formula and section F for numerical simulations to test it.) When applied to an independent sample, the test accuracy of the model depends on the between-sample heterogeneity (as discussed in Section “Testing a Model’s Accuracy in an Independent Validation Sample: Heterogeneity in the Population, Causing between-Sample Heterogeneity”). For a sample consisting of two homogeneous subsamples s_1_ and s_2_, the accuracy drop is in the range 70–90% approximately given by: Δ*acc* ≈ 26% ×^10^log((1 + *f*_12_)/2), as compared to the accuracy in sample s_1_ or sample s_2_ alone.

These formulas can be extended to samples with *H*-fold within-sample heterogeneity (*H* ≥ 2, see Figure 3C) leading to larger drops in accuracy. The reader is referred to the Datasheet S1 in Supplementary Material for the corresponding formulas. Note that thus far we treated the heterogeneity as being discrete (*H* = 2,3,…). While this may be a good description for disease heterogeneity related to, e.g., gender, in general the heterogeneity will probably have a continuous nature. For instance, first-episode versus chronic is not a hard cut, but is described by the continuous parameter illness duration. This can be incorporated in the formulas by allowing *H* to assume non-integer values.

#### 2. Variation

##### 2a. Non-Disease-Related Heterogeneity

Heterogeneity with respect to factors not related to the disease gives rise to variation in brain measures (Figure 2B-2). This biological variation is present in both healthy subjects and patients. The more heterogeneous a sample is, the larger the variation in feature values. Examples are as follows: including males and females (as opposed to single-gender); including subjects with a wide age or IQ range; genetic background (but for genetic disease-related genetic factors, see Heterogeneity of the Disease). Relaxing the inclusion criteria of a study will thus increase the variation in feature values {*x_*i*_*}.

###### Matching of subjects with respect to nuisance variables: Confounding the classifier

A special form of increased variation arises when subjects are not well-matched with respect to nuisance variables. If the distributions of factors such as age and gender are not well-matched between train and test samples, (parts of) the classifier’s output distribution (*y*) may shift, causing a change in the sensitivity/specificity balance. Within-sample mismatch with respect to nuisance variables between patients and controls will confound the classifier: part of the discrimination between the two groups will be based on brain abnormalities unrelated to the disease. For instance, if the patients are on average older than the controls, brain volume decreases related to normal aging may be used by the classifier to separate the groups. When applied to a test sample with a different demographic composition, this part of the model will not contribute to the separation of the groups, and the effect size will be lowered by a factor *f* < 1 (see Testing a Model’s Accuracy in an Independent Validation Sample: Heterogeneity in the Population, Causing between-Sample Heterogeneity).

##### 2b. Measurement Noise

The second factor that increases the variation in feature values {*x_*i*_*} is measurement noise (Figure 2B-3). Features are derived from measurements done by, e.g., an MRI scanner, which inherently involves noise. Random noise, such as, e.g., physiologic and electronic noise and noise due to subject movements, leads to an uncertainty in the feature values. Systematic (and interaction) effects, due to differences in, e.g., scanner brand and type, field strength, and acquisition protocols, play a role when two or more samples with different origin are combined, e.g., in a train/test study. These effects can result in biased sensitivity/specificity (e.g., when certain parts of the brain show up differently between two scanners) or in changes in the prediction accuracy (which will be lower for noisier scans, but could even go up if test scans were acquired with less noise, e.g., on a scanner with higher field strength).

While variation due to inclusion of biologically heterogeneous subjects (see Non-Disease-Related Heterogeneity) and Section “measurement noise” is completely different in nature, their effects on the ML accuracy run via the same channel: increased variation in the features {*x_*i*_*}, which is carried over to the variance of the prediction model’s output: σ*_*y*_*. The total variance is given by: σ^2^*_*y*_* = σ^2^_BIOL_ + σ^2^_EXP_. Lower or higher variance (σ*_*y*_*) in the test set as compared to the training set can cause increases or decreases (respectively) in the test accuracy, according to the same formula as used in Section “Testing a Model’s Accuracy in an Independent Validation Sample: Heterogeneity in the Population, Causing between-Sample Heterogeneity,” since it is determined by the effect size *d*_ML_ = *f*Δ*y*/σ*_*y*_*. A twice as large noise will have the same effect as a 50% overlap.

#### 3. Sampling Effects (Finite *N*)

In samples of finite size, random variations influence both the modeling (training phase) and the testing. Two effects play a role here.

##### 3a. Train/Test Case

First, if we assume that a theoretical, population-based, model exists, then in practice a model will depend on the actual training sample taken from “the population.” Differences in the amount and kind of heterogeneity in the selected subjects will cause both differences in prediction accuracy and “positioning” of the model [optimal separation hyperplane (OSH)] with respect to the population-based model. Models “further away” from this population-based model will more likely perform worse (i.e., producing lower accuracies) in a second, independent test sample from the same population. The accuracy in test samples is, thus, hit twice by the sampling effect: once due to fluctuations in the test sample’s composition itself (having a lucky or an unlucky drawing of subjects with respect to the population distribution) and a second time because of the composition of the training sample, on which the model was built (relative position of the two samples). This effect is larger in smaller (training and testing) samples. The observed accuracies officially follow a binomial distribution, but can be approximated by a normal distribution with mean *acc*_true_ and an SD of √(*acc*_true_ × (100%−*acc*_true_)/*N*). For an *acc*_true_ of 80%, SD = 40%/√*N*, thus in a sample of *N* = 50 (25 + 25 per group), SD = 5.7%, giving a chance of 95% that the observed *acc* lies between 68.6 and 91.4%. For four times as large sample, *N* = 200, this range is 74.3−85.7%. Note that the sampling effect, thus, does not systematically lower the accuracy, but that it gives rise to variation in it. It is true, however, that the lower the *N*, the larger the chance of observing a low accuracy (as well as a high accuracy).

##### 3b. Cross-Validation Case

Second, if performance is tested within the training set, CV will induce small perturbations in this set during each step of the leave-one(or more)-out procedure, accounting for further fluctuations around the “theoretical” OSH (Figure 2B-3). The effect on the (CV) accuracy is difficult to estimate, since it will depend on the type of classifier used. For example, a support vector machine (SVM) model is based on only part of the training subjects [the so-called support vectors (SV)], who are recognized as lying close to the OSH during the training phase. Leaving-out a non-SV subject will not influence the model, while leaving out a SV subject probably will change the placement of the OSH and, thus, the classification of subjects nearby the OSH. If the number of support vectors (*N*_SV_) is known, a reasonable estimate of the SD of the accuracy could be made by the formula for SD in the previous paragraph, using *N*_SV_ instead of *N*.

Note that sampling effects induce spread in accuracy, but not a reduction of it *per se* (although the spread distribution becomes asymmetric for population-based accuracies above 50%, the expectation value of the accuracy in a sample always equals the accuracy in the population). However, since the spread is larger for lower *N* and (accidently) low accuracies are less likely to be published, the sampling effect may add to the on average higher accuracies in low-*N* studies.

An illustration of the sampling effect as a function of training sample size *N* can be found in Ref. (11). In their Figure 3, the rise of the mean accuracy with *N* reflects a training sample-based OSH that lies closer to the population-based OSH [see Train/Test Case]. The lower spread in observed accuracies with increasing *N* (light blue circles) reflects the decreasing SD [see Cross-Validation Case]. Of course, the fact that these effects occur is a reflection of the disease heterogeneity (see Heterogeneity of the Disease) and biological variability (see Variation) present in the sample: without variability sampling effects do not play a role.

#### 4. Training: From Gold to Silver Standard

Last but not least, in a supervised learning study, the quality of the expert labeling of the subjects in the training set (and, in fact, in the test set too) influences the highest possible accuracy. Unfortunately, especially for prediction problems in psychiatry, the reliability of the experts may not always be that high [see Ref. (22), for a study on reliability of DSM-5 diagnoses, using interclass kappa (23)]. This means that, in many cases, we have, unfortunately, not a gold, but rather a silver standard (Figure 2B-4). The quality of the standard is different for different classification problems, and may be close to 0 in certain situations, but for diagnosis of SZ and bipolar disorder using SCID-I inter-rater reliabilities of 80–94% has been found (24) and using DSM-5 interclass kappa’s of 0.46–0.56 (22). In general, a drop of ~10% can safely be assumed. Of course, 100% accurate training models can still be obtained, but there is a hidden inaccuracy due to subjects wrongly labeled by the expert. While this inaccuracy goes unnoticed in the training results, it will at any rate be revealed in the test phase, either by CV or in a test sample. Although mislabeling by the expert may be linked to the more difficult cases, ignoring this leads to a simple formula for a two-class test case: *acc* = κ × *acc*_0_ + (1 − κ)/2, which is smaller than the true accuracy (*acc*_0_) for *acc*_0_ > 50%; κ is the interclass kappa, which, for a two-class/two-rater case can be related to the fraction of cases mislabeled by the experts: (1 − κ)/2. This formula could even be extended for estimating the loss of accuracy in the CV in the training sample, but this depends on the type of classifier. The CV procedure is hit twice by errors in the expert labeling: when the left-out subject is predicted (as described above), but also when the model is built. If all subjects influence the model, the effect is about the same as in the test phase. If, however, as e.g., SVM, only part of the subjects (*N*_SV_) actually influences the model, a mislabeled non-SV subject has no influence. On the other hand, probably the subjects close to the separation border, the SVs, are also the ones that are most difficult to be classified by the expert.

#### Summary

To summarize (see Figures 4A–E), imperfection of the gold standard (Figure 4A) will lower the ceiling of the training accuracy (ideally, 100%) in the training set [silver standard (Figure 4B)]. (Within-sample) disease-pattern heterogeneity will introduce the impossibility to capture all discriminating brain abnormalities needed for the classification of all subjects, thus reducing the accuracy (Figure 4C). Finite sample sizes and biological variation and measurement noise will lower the (*k*-fold; leave-one-out) CV accuracy further (Figure 4D). In an independent test sample, when the model is applied to a new data set, this negative effect of (between-sample) heterogeneity on the prediction accuracy will be magnified, and sampling effects will cause further spread in the test accuracy (Figure 4E). The loss will depend on the overlap in discriminative features between train and test sample, or, the between-sample disease-pattern heterogeneity.

#### Relationship between the Heterogeneity Factor “f” and the (Feature) Weight Vectors, Cos(θ): The Angle of Heterogeneity

In linear models, such as, e.g., the linear SVM, the model can be represented by a set of coefficients, β, or weight vector *w*, indicating the features’ relative importance. The decision value *y* is the dot product of this vector and the feature vector: *y* = *w*^T^.*x* (offset *b* is not relevant here). When comparing two models, the dot product of the (normalized) weight vectors can be informative: it provides a summary statistic of the comparability of the two models. It can be shown (see Datasheet S1 in Supplementary Material) that the disease heterogeneity factor *f* = cos(θ), the dot product of the normalized weight vectors. θ is the angle between the two vectors (or, equivalently, the separating hyperplanes): the angle of heterogeneity. In this context, it should be noted that disease heterogeneity can arise in two forms. Thus far we split discriminative features in a part shared by subgroups of patients and in features specific to each of the subgroups (and, thus, absent in the other subgroups). It is, however, also possible that certain features are discriminative in more than one subgroup, but in different directions: for instance, a piece of the cortex that is either too thin or too thick could be disadvantageous and, thus, related to having the disease. Such manifestations of heterogeneity of the disease could lead to angles larger than 90° and, thus, negative *f*. The values *f* can assume are, thus, between −1 and +1. While *f*, or cos(θ), thus provide an indication of the global comparability between two models, detailed information needs to be obtained by comparison on a weight-by-weight basis, e.g., by comparing projections of the weight vectors on brain maps.

### Application to the Published sMRI-ML Schizophrenia Studies

Figure 1 shows the derived effects in relation to the published sMRI-ML studies in SZ.

The heterogeneous-sample model has been “fitted” to the data points representing CV studies, using parameter values of (*d*_0_ = 2.00; *f*_12_ = 0; *N* = *H* × *N*_0_; *N*_0_ = 50). Note that this “fit” is descriptive (i.e., no rigorous optimization of the goodness-of-fit with optimal parameter estimates and confidence intervals was carried out): the line is an indication of how the fall of the accuracy with sample size can be explained by increasing within-sample heterogeneity. We assumed constant values for the homogeneous-sample ML effect size *d*_0_, the (within-sample) heterogeneity factor *f*_12_, and the homogeneous-subsample size *N*_0_, but these values can, of course, vary between the studies. For smaller samples (up to *N*≈60), the accuracy seems to lie at a plateau (~90%). This could indicate that researchers have been able to stretch the maximum sample size that allows the inclusion of patients with homogeneous disease-related brain abnormalities. The ~90% ceiling of the accuracy probably reflects the maximum possible accuracy that can be obtained with imperfect gold standard (see Training: From Gold to Silver Standard).

Four studies tested their HC/SZ classification model in an independent validation sample. Two small studies obtained test accuracies that were even higher than the train accuracies, which is probably due to the effect of sampling [having a lucky drawing in the test sample; Section “Sampling Effects (Finite *N*)”], which is larger for low *N*. The 7% jump in accuracy (from 77% in the training set to 84% in the test set) found by Kawasaki et al. (25) is of the order of the uncertainty in the accuracy, SD = 5–7%, for these sample sizes (*N* = 30 + 30 and *N* = 16 + 16, respectively). The other possible cause, better image quality in the test set, seems not applicable here, since both sets were drawn from the same cohort. The study by Iwabuchi et al. (19), on the other hand, clearly shows the effect of scan quality: Using the same sample, they built two classification models: one based on 3-T scans and one based on 7-T scans. While increasing field strength does not automatically lead to improved scan quality, higher resolution or better contrast-to-noise ratio is usually obtained. Indeed, the accuracy increased from 67% in the 3-T models to 77% in the 7-T models. (Note that sampling effects do not play a role here, since the same subjects were used for both models.)

Two other (smaller-sized) studies displayed a large drop (>10%) of accuracy in the test sample as compared to the train sample; note that the *N*/2 = 66 study used a test set acquired at different field strength (3 T versus 1.5 T). This can be explained by the fact that low-*N* training samples can be more homogeneous, giving rise to (i) the higher CV accuracies (>88%) seen here [see (Cross-Validation) Accuracy of the Model: Heterogeneity within Larger Training Samples] and (ii) a larger mutual, or between-sample, heterogeneity with the test sample, yielding larger drops in accuracy (see Testing a Model’s Accuracy in an Independent Validation Sample: Heterogeneity in the Population, Causing between-Sample Heterogeneity). Of course, sampling effects could also add to the large differences here. The only large train/test study (*N*/2 = 120) showed almost no (1%) drop in accuracy between train and test sample. This is in agreement with the theory that larger studies automatically capture more (within-sample) disease heterogeneity and, thus, better generalize – at the cost of lower accuracy. For three train–test studies, the overlap in discriminative features could be estimated, with relatively low values of *f*_12_ = 0.6, for the smaller studies, and a high value for the larger study: *f*_12_ = 0.95.

The study by Nieuwenhuis et al. (11) reported a full model (presented in Figure 1) and two sub-models. The first sub-model excluded the striatum, known for being affected by typical antipsychotics (26), from the feature set. Half of the patients in the training sample were on typical antipsychotics, and as could be seen from their Figure 2, the discrimination between patients and controls was in part based on gray matter differences in the striatum. Excluding the striatum leads to a 4% reduction in classification accuracy. This drop could be attributed to less discriminative information being present in the sample and, thus, a drop in “separation strength” Δ*y* (see Heterogeneity of the Disease) and ML effect size *d*_ML_ and accuracy for the discovery sample. However, since hardly any patients (8%) of the validation sample were on typical antipsychotics, the part of the model based on the medicated striatum was of no discriminating value for this sample and, thus, nothing changed for the validation performance (+0.2%). [The striatum features were located in the s_1_-petal (Figure 3A).] The second sub-model was trained on the top-10% features with the largest absolute weights. The CV accuracy of this model increased to 86.8%, probably because the other 90% of the features did not contribute much to the discrimination (separation strength Δ*y* hardly changed) but did add quite some noise: the spread in predictions, σ^2^, decreased and the ML effect size *d*_ML_, thus, increased. This model, however, was apparently more tuned (“overfitted”) to the specific constitution of the training sample with respect to its disease heterogeneity (i.e., the petals in Figure 3 had become relatively large as compared to the core), and thus the improved prediction accuracy was not found again in the validation sample, which had an almost unchanged accuracy of 69.1%.

## Discussion

In this work, we examined the various sources that influence the performance of ML classification and prediction studies in psychiatric neuroimaging. The published studies on the prediction of SZ using sMRI display a wide variation in classification accuracies. From a plot of accuracy versus sample size (Figure 1), we showed that the accuracy of these classification studies drops from a plateau at about 90% in smaller samples (*N*/2 < ~60) to (below) 70% for studies with larger *N*. A simple heterogeneous-sample model was able to follow this drop in (maximum) accuracy with increasing sample size. Smaller studies are better capable of including homogeneous samples, which allow for the discovery of discriminative brain features that apply to all patients, yielding models with high accuracy. Larger studies inevitably need to relax the inclusion criteria, yielding heterogeneous samples in which no discriminative pattern of brain abnormalities can be found that is shared by all patients. As a result, only part of the subjects will be correctly classified, resulting in lower accuracy.

Application of these classification models to independent validation samples allows for testing their generalizability. From the few studies that performed such a test, it was observed that accuracy can drop as much as 10–15% or even can increase for the smaller studies. The only study with large train/test samples showed a much more stable accuracy (a drop of only 1%). Patients in a test sample will most likely display a different pattern of brain abnormalities as compared to those in the training set, i.e., the two samples are mutually heterogeneous. The accuracy in the replication sample will be (much) lower, depending on the amount of shared features between the two samples. The drop in accuracy will presumably be smaller for studies with a large training sample, since it will automatically cover more disease features from the set of all possible features.

An additional advantage of larger studies is that they are less prone to sampling effects. The larger variability in accuracy, which is observed for smaller studies, could be explained by lucky/unlucky drawings from the patient population.

Summarizing, sample size influences the trade-off between accuracy and generalizability. Smaller, homogeneous, samples are able to produce classification models with high accuracy, at the cost of low generalizability, whereas larger, heterogeneous samples produce models that better generalize, but at the cost of lower accuracy. We argue here that, with the current approaches, high accuracy cannot be reached in larger, heterogeneous samples – in psychiatry. From an evaluation of the ML literature on neurological diseases (10), it is noted that ML studies on Alzheimer’s disease seem to reach high accuracy from small *N* till very large *N*. This may be attributed to the more precise characterization of this disease, thus leading to a more homogeneous population and thus samples. Studies on mild cognitive impairment, on the other hand, do show a substantial drop in accuracy with *N*, probably because this class of patients is less well defined, and thus leading to increased (within-sample) heterogeneity.

The spread in accuracy seen in first-episode SZ (FE-SZ) ML studies could have two causes. On the one hand, the FE-SZ class of patients does show some variability in presentation and clinical course, but is, compared to a chronic/mixed group of patients, more homogeneous [shorter duration of illness; less variability in age and disease course; less (variation in) (cumulative) medication (dose); etc.] allowing for higher accuracy. On the other hand, the disease effects in the brain may be smaller in these patients than in chronic patients, lowering the separability of the groups. Both effects influence the ML effect size and, thus, the model’s performance. Spread in inclusion criteria between studies, for instance, with respect to illness duration, can have led to differences in effect sizes between the FE-SZ studies. Given the difficulty to include large number of FE-SZ subjects, all FE-SZ ML studies were relatively small (*N*/2 < 65). Sampling effects could also contribute to the large variation in accuracies, for these relatively low numbers of subjects.

There are many possible sources of heterogeneity. Psychiatric disorders may be divided into subtypes (see, e.g., Ref. (21) for SZ). Within “homogeneous subtypes,” the disease status of patients plays a role: illness severity (outcome) and course (age-of-onset and illness duration; number of psychoses, etc.); medication use [type and (cumulative) dose], etc. Furthermore, even a “clinically” homogeneous patient group may show heterogeneity in their underlying brain abnormalities, because, e.g., different (disease-related) genetic factors may cause different biological pathways to the same (subtype of the) disease. Nuisance variables also further increase a sample’s heterogeneity, because of (normal) variation in brain tissue properties related to age, gender, IQ, and so on. Apart from all these biological factors that influence “true” heterogeneity, experimental heterogeneity is introduced by, e.g., scanner effects.

Although we can explain the observed accuracy distributions both qualitatively and quantitatively to large extent by (disease) heterogeneity and sampling effects, it should be noted that there are other possible explanations as well. Lower (CV) accuracy could simply reflect a worse model due to reasons, such as poorer quality of the input data, for instance, due to scan quality (acquisition, subject motion), image preprocessing, or choices made regarding the kind of features (e.g., high-resolution gray matter volumes may be more discriminative than mean-FA values in the fiber tracts). Other possible causes include a less fortunate selection of features or suboptimal modeling methods (e.g., choice of ML type or parameter settings).

While from these observations the picture may arise that studies employing both large (training) samples and independent validation samples are most powerful and informative, studies with smaller *N* are as useful for the understanding of the biological background of the disease for several reasons. If a small *N* study included an application of the model to an independent replication sample, the drop in accuracy carries information about the (mutual) homogeneity of the sample. In fact, if the study has the disposal of two independent samples, two models should be trained on each sample separately, which should be tested on the other sample. This cross-sample application provides information about the within- and between-samples heterogeneity and allows for comparison of the separating brain patterns, yielding shared and sample-specific discriminative brain features. In a later stage, the results of studies could be combined for the same purpose: mapping the variability (in populations) of underlying brain patterns for classification and prediction of psychiatric disorders. For example, ML brain patterns from medication naïve patients could be compared to those from medicated first-episode patients. Special populations for which it is difficult to acquire large samples can provide biomarker information that is specific for that population. To get the most out of such samples, both as a single study and in possible later multi/cross-center studies, it is important to have as much as possible demographic and clinical information available.

In order to interpret the published studies and value them for use in cross-study application, it is thus important that details of the analysis and results are reported. For instance, in SVM studies, the number of subjects the model is based on, i.e., the *N*_SV_, could be provided. As an example, the SZ classification model by Nieuwenhuis et al. (11) was based on *N*_SV_ = 257 out of *N* = 294 subjects in total. The relative large number of SVs (87%) could reflect the large heterogeneity of the training sample. Likewise, in a (M)LDA approach (see, e.g., Ref. (27)), the number of eigenvectors used in the model could be reported.

### Limitations

In this work, we described the factors influencing ML model performance qualitatively and quantitatively. For the quantitative description, there was only room here to treat the most elementary form of sample heterogeneity and its effect on linear prediction models. We believe, however, that it covers the principle of disease heterogeneity to “first-order approximation.” The theory should be extended to include refined descriptions of sample heterogeneity and the effects in other ML setups: non-linear models, more than two classes, prediction of continuous measures, such as disease course (outcome) and so on. The ML effect size could be extended beyond the discrete, binary, case. Systematic (scanner etc.) effects were ignored, which will influence sensitivity and specificity in a different way. The implications of sample heterogeneity were mostly discussed within the framework of linear classifiers, and in particular the linear SVM. The theory should be broadened to other types of ML such as Gaussian Processes (28) and (M)LDA (27), and non-linear classifiers. Non-linear classifiers might be better capable of modeling the heterogeneity, but are more prone to overfitting, thus possibly reaching higher accuracies in the training sample, but which are less generalizable to other samples. However, using much larger (multicenter) samples may (partly) overcome this drawback. (Group-level-based) feature selection may reduce heterogeneity of the features, while using lower-dimensional brain features, e.g., by taking ROI-based measures instead of voxel/vertex-based measures or by applying principal component analysis (PCA) to the high-dimensional brain data, could have the same effect. While this may increase the robustness of the models, they will less well incorporate the variation in disease-related brain abnormalities, thus, probably not be able to reach high generalization performance.

In conclusion, the wide variation in observed prediction accuracy in this young field of research is an indication that the ML models are built on samples that are mutually very different. Disease heterogeneity, (normal) biological variation, noise and sampling effects, and imperfect expert labeling influence the results. Sample size and observed accuracy can be translated into information about the within- and between-sample heterogeneity, which, in turn, could be interpreted in terms of the sample characteristics, if provided. The meaning of a study’s accuracy is limited if it cannot be connected to the study design and characteristics of the sample. Pursuing a high accuracy should not be a goal in its own if we aim to increase the knowledge about the biological background of the disease. Furthermore, one should be cautious with statements about the potential clinical use of some prediction model, even if it yielded high prediction accuracy. Accuracy is a relevant measure, but only in combination with a detailed description of the sample and design of the study it gives us valuable information.

For the next generation of ML studies in psychiatric imaging to be as fruitful as possible, we would recommend the following:

1. The report of more details of the sample(s) and ML analysis.
  (a) Regarding the sample(s): as much as possible information about the sample should be provided: demographic and clinical parameters: distribution of gender, ethnicity, (range and mean, SD of) age, IQ, socioeconomic status, geographical background, etc.; duration of illness/age of onset, (dose and type of) medication, scores of functioning and outcome, and so on. Furthermore, neuroimaging parameters, such as MRI acquisition and preprocessing details should be provided.
  (b) Regarding the modeling: (i) inputs and settings: these details include type and number of features in the (final) model; parameter settings (e.g., C in linear SVM); (ii) relevant properties of the resulting model, when possible, such as (pictures of) weight vectors (weight maps) and their significance, and, in SVM, e.g., the *N*_SV_.
  (c) Regarding the model’s performance: balanced (or total) accuracy, sensitivity and specificity (or, equivalently, positive and negative predicted values), and the area under the curve (AUC) from a receiver operating curve (ROC) analysis, that, in itself, provides more insight into the balance between sensitivity and specificity at different thresholds. Furthermore, the resulting effect size (*d*_ML_), calculated from the separation strength (Δ*y*) and SDs of the mean *y*, for each of the classes (groups) should be reported, as well as the effect size calculated from the AUC (see Datasheet S1 in Supplementary Material section G for formulas and a Matlab script). For ML algorithms that do not produce “about-normally” distributed output (e.g., voting), the ML effect size could be estimated by calculating 2 × Φ^−1^(*acc*). Bootstrap analyses enable the estimation of uncertainties in the estimated *d*_ML_’s and accuracies and resulting *p*-values and confidence intervals.
2. Additional modeling. The performance of the (final) model could be improved by applying model averaging, such as (su)bagging, lowering the variance of the model’s output (29). Sub-modelings, e.g., with different selections of features, related to the parameters described in point 1, could shed more light on the relationship between certain features and subgroups of subjects. An example is the modeling of males and females separately. Apart from these extra models, the performance of the final model on these subgroups itself already provides insight into possible interactions between, e.g., gender and classification.
3. Use large (single-center) samples to build classification models: they automatically cover more variation in the disease features (and are less influenced by accidental variations and noise) and, thus, more robust (for application to other samples).
4. Validation. If possible, always use a training sample and an *independent* replication sample. Independency here means that the subjects were at least not acquired as part of the first study.
5. Apply cross-center validation. Models built in one site could be tested in the other, and vice versa. This is an extension of point 4. Do not be afraid of substantial losses in accuracy: they carry information about the overlap in disease features. Further extending this line of thought is the possibility to build prediction models from multicenter data; technical (scanner) and clinical (diagnostic) differences may somewhat degrade the performance, but the shared disease factors will survive (30). Recently, multicenter consortia have recently been initiated to investigate the possibility of translating neuroimaging findings into clinical practice (IMAGEMEND^1^ PRONIA^2^ and PSYSCAN^3^).

## Author Contributions

HS and RK made substantial contributions to the conception (RK) and design (HS) of the work and the analysis and interpretation (HS) of the data. HS drafted the work and RK revised it critically. HS and RK gave final approval of the manuscript. HS and RK agree to be accountable for all aspects of the work.

## Conflict of Interest Statement

The authors declare that the research was conducted in the absence of any commercial or financial relationships that could be construed as a potential conflict of interest.

## Footnotes

^1^http://www.imagemend.eu

^2^www.pronia.eu

^3^www.psyscan.eu

## References

[1] Kambeitz J Kambeitz-Ilankovic L Leucht S Wood S Davatzikos C Malchow B Detecting neuroimaging biomarkers for schizophrenia: a meta-analysis of multivariate pattern recognition studies. Neuropsychopharmacology (2015) 40:1742–51. 10.1038/npp.2015.22 25601228 PMC4915258

[2] Segovia F Holt R Spencer M Górriz JM Ramírez J Puntonet CG Identifying endophenotypes of autism: a multivariate approach. Front Comput Neurosci (2014) 8:60. 10.3389/fncom.2014.00060 24936183 PMC4047979

[3] Hart H Chantiluke K Cubillo AI Smith AB Simmons A Brammer MJ Pattern classification of response inhibition in ADHD: toward the development of neurobiological markers for ADHD. Hum Brain Mapp (2014) 35:3083–94. 10.1002/hbm.22386 24123508 PMC4190683

[4] Schnack HG Nieuwenhuis M van Haren NE Abramovic L Scheewe TW Brouwer RM Can structural MRI aid in clinical classification? A machine learning study in two independent samples of patients with schizophrenia, bipolar disorder and healthy subjects. Neuroimage (2014) 84:299–306. 10.1016/j.neuroimage.2013.08.053 24004694

[5] Mourao-Miranda J Reinders AA Rocha-Rego V Lappin J Rondina J Morgan C Individualized prediction of illness course at the first psychotic episode: a support vector machine MRI study. Psychol Med (2012) 42:1037–47. 10.1017/S0033291711002005 22059690 PMC3315786

[6] Kambeitz-Ilankovic L Meisenzahl EM Cabral C von Saldern S Kambeitz J Falkai P Prediction of outcome in the psychosis prodrome using neuroanatomical pattern classification. Schizophr Res (2015). 10.1016/j.schres.2015.03.005 25819936

[7] Koutsouleris N Davatzikos C Bottlender R Patschurek-Kliche K Scheuerecker J Decker P Early recognition and disease prediction in the at-risk mental states for psychosis using neurocognitive pattern classification. Schizophr Bull (2012) 38:1200–15. 10.1093/schbul/sbr037 21576280 PMC3494049

[8] Borgwardt S Koutsouleris N Aston J Studerus E Smieskova R Riecher-Rössler A Distinguishing prodromal from first-episode psychosis using neuroanatomical single-subject pattern recognition. Schizophr Bull (2013) 39:1105–14. 10.1093/schbul/sbs095 22969150 PMC3756775

[9] Zarogianni E Moorhead TW Lawrie SM. Towards the identification of imaging biomarkers in schizophrenia, using multivariate pattern classification at a single-subject level. Neuroimage Clin (2013) 3:279–89. 10.1016/j.nicl.2013.09.003 24273713 PMC3814947

[10] Orrù G Pettersson-Yeo W Marquand AF Sartori G Mechelli A Using support vector machine to identify imaging biomarkers of neurological and psychiatric disease: a critical review. Neurosci Biobehav Rev (2012) 36:1140–52. 10.1016/j.neubiorev.2012.01.004 22305994

[11] Nieuwenhuis M van Haren NE Hulshoff Pol HE Cahn W Kahn RS Schnack HG. Classification of schizophrenia patients and healthy controls from structural MRI scans in two large independent samples. Neuroimage (2012) 61:606–12. 10.1016/j.neuroimage.2012.03.079 22507227

[12] Krystall JH State MW. Psychiatric disorders: diagnosis to therapy. Cell (2014) 157:201–14. 10.1016/j.cell.2014.02.042 24679536 PMC4104191

[13] Zhang T Koutsouleris N Meisenzahl E Davatzikos C. Heterogeneity of structural brain changes in subtypes of schizophrenia revealed using magnetic resonance imaging pattern analysis. Schizophr Bull (2015) 41:74–84. 10.1093/schbul/sbu136 25261565 PMC4266302

[14] Janousova E Schwarz D Kasparek T. Combining various types of classifiers and features extracted from magnetic resonance imaging data in schizophrenia recognition. Psychiatry Res (2015) 232:237–49. 10.1016/j.pscychresns.2015.03.004 25912090

[15] Castro E Martínez-Ramón M Pearlson G Sui J Calhoun VD. Characterization of groups using composite kernels and multi-source fMRI analysis data: application to schizophrenia. Neuroimage (2011) 58:526–36. 10.1016/j.neuroimage.2011.06.044 21723948 PMC3242731

[16] Pohl KM Sabuncu MR. A unified framework for MR based disease classification. Inf Process Med Imaging (2009) 21:300–13. 10.1007/978-3-642-02498-6_25 19694272 PMC2854674

[17] Schnack HG van Haren NEM Nieuwenhuis M Hulshoff Pol HE Cahn W Kahn RS Accelerated brain-aging in schizophrenia: a longitudinal pattern recognition study. Am J Psychiatry (2016). 10.1176/appi.ajp.2015.15070922 26917166

[18] Takayanagi Y Kawasaki Y Nakamura K Takahashi T Orikabe L Toyoda E Differentiation of first-episode schizophrenia patients from healthy controls using ROI-based multiple structural brain variables. Prog Neuropsychopharmacol Biol Psychiatry (2010) 34:10–7. 10.1016/j.pnpbp.2009.09.004 19751790

[19] Iwabuchi SJ Liddle PF Palaniyappan L. Clinical utility of machine-learning approaches in schizophrenia: improving diagnostic confidence for translational neuroimaging. Front Psychiatry (2013) 4:95. 10.3389/fpsyt.2013.00095 24009589 PMC3756305

[20] Cohen. Statistical Power Analysis for the Behavioral Sciences. 2nd ed Hillsdale, NJ: Lawrence Erlbaum Associates (1988).

[21] Derks EM Allardyce J Boks MP Vermunt JK Hijman R Ophoff RA Kraepelin was right: a latent class analysis of symptom dimensions in patients and controls. Schizophr Bull (2012) 38:495–505. 10.1093/schbul/sbq103 20864620 PMC3329975

[22] Regier DA Narrow WE Clarke DE Kraemer HC Kuramoto SJ Kuhl EA DSM-5 field trials in the United States and Canada, Part II: test-retest reliability of selected categorical diagnoses. Am J Psychiatry (2013) 170:59–70. 10.1176/appi.ajp.2012.12070999 23111466

[23] Fleiss JL Spitzer RL Endicott J Cohen J Quantification of agreement in multiple psychiatric diagnosis. Arch Gen Psychiatry (1972) 26:168–71. 10.1001/archpsyc.1972.01750200072015 4551259

[24] Skre I Onstad S Torgersen S Kringlen E. High interrater reliability for the structured clinical interview for DSM-III-R axis I (SCID-I). Acta Psychiatr Scand (1991) 84:167–73. 10.1111/j.1600-0447.1991.tb03123.x 1950612

[25] Kawasaki Y Suzuki M Kherif F Takahashi T Zhou SY Nakamura K Multivariate voxel-based morphometry successfully differentiates schizophrenia patients from healthy controls. Neuroimage (2007) 34:235–42. 10.1016/j.neuroimage.2006.08.018 17045492

[26] Smieskova R Fusar-Poli P Allen P Bendfeldt K Stieglitz RD Drewe J The effects of antipsychotics on the brain: what have we learnt from structural imaging of schizophrenia?—a systematic review. Curr Pharm Des (2009) 15:2535–49. 19689326 10.2174/138161209788957456

[27] Kasparek T Thomaz CE Sato JR Schwarz D Janousova E Marecek R Maximum-uncertainty linear discrimination analysis of first-episode schizophrenia subjects. Psychiatry Res (2011) 191:174–81. 10.1016/j.pscychresns.2010.09.016 21295452

[28] Mourão-Miranda J Almeida JR Hassel S de Oliveira L Versace A Marquand AF Pattern recognition analyses of brain activation elicited by happy and neutral faces in unipolar and bipolar depression. Bipolar Disord (2012) 14:451–60. 10.1111/j.1399-5618.2012.01019.x 22631624 PMC3510302

[29] Andonova S Elisseeff A Evgeniou T Pontil M A simple algorithm to learn stable machines. Proceedings of the 15th European Conference on Artificial Intelligence (ECAI) 2002 (2002). p. 513–7.

[30] Dluhoš P Schwarz D Cahn W van Haren N Kahn R Kašpárek T Multi-center machine learning in imaging psychiatry: a meta-model approach. (in review). 10.1016/j.neuroimage.2017.03.027 28428048

