# Supplementary material for: Detecting Neuroimaging Biomarkers for Psychiatric Disorders: Sample Size Matters
Source: Front Psychiatry. 2016 Mar 31;7:50. doi: 10.3389/fpsyt.2016.00050 (PMC4814515; doi:10.3389/fpsyt.2016.00050)
Supplement: Supplementary file 1 [file Data_Sheet_1.PDF]

**APPENDIX – Derivation of the heterogeneous sample model**

**A**. Consider a homogeneous sample of patients and healthy control subjects. The sample ($s_1$) is taken from a population of patients with possible disease heterogeneity, but we assume that this sample, due to its inclusion criteria, is homogeneous – in its brain features (and probably in symptoms too). (Figure 3a, single blue petal). For the sake of simplicity we assume that the numbers of patients and controls are equal, $N$ (note that in ML studies balanced group sizes are preferred). A ML model ($M_1$) is built from the sample's feature set $\{x\}_1$, resulting in a weight vector $w_1$, that describes the relative weight and direction (towards healthy (value -1) or ill (value +1)) each feature has on the classification of a subject. The predicted class for subject $j$ depends on the sign of the following product: $y_j = w^T.x_j + b$ (in the following we will omit the offset $b$, regulating the bias between sensitivity and specificity). The model is tested using (leave-one-out) cross-validation (LOOCV), where the model is applied to the training sample itself, but with sequentially each subject left out in the training phase, which is then used to test the model: $M_1(s_1)$ -> accuracy (%). From a sample that is not too small and with patients that are homogeneous with respect to the underlying brain abnormalities, a classification model can be built with high LOOCV prediction accuracies, provided that the feature set includes the relevant, i.e., discriminative, features. Measurement noise and imperfect expert labeling will result in an accuracy lower than 100% though.

As pointed out in the ML effect size section (see Fig. 2-A), the means and variation of the features is transformed, by $M_1$, to a mean $y$ ± standard deviation ($\sigma_y$, Fig. 2-A2), for each of the classes. Assuming a normal distribution, the resulting ML effect size, $d_{ML} = \Delta y/\sigma_y$, can be used to calculated the fraction of subjects that can be correctly classified if a threshold is placed between the two distributions (Fig. 2-A3): $acc = \Phi(d_{ML}/2)$, with $\Phi(.)$ the cumulative normal distribution. In a formula:

$M_1$ on $s_1$: $\quad \Delta y \quad \pm \quad \sigma_y , \quad d_{ML} = \Delta y/\sigma_y$ (1)

**B**. Now, we apply this model $M_1$ to an independent test sample, $s_2$. To keep the notation simple, we assume that the numbers of subjects and discriminating features are equal between the two samples. The second sample shares part of the discriminating features with sample 1, but each sample has included patients with brain abnormalities specific to that sample (see Fig. 3a, yellow petal). If we denote the size of the discriminative feature sets by $A_1=A_2=A$, and the size of the overlapping part (i.e., the shared features) by $A_{12}$, we can define the fraction of shared features by $f=f_{12}= A_{12}/A$. (This definition can be extended to more complex shared and sample-specific feature sets, and related to the dot product of the weight vectors of the models built on the separate samples, $M_1$ and $M_2$, see section E.) If $M_1$ is applied to $s_2$, only the shared part of the features will contribute to the separation of the two classes in $s_2$: the $s_1$-specific part will have no effect on $s_2$ and the discriminative features specific to $s_2$ are not part of $M_1$. In a formula:

$M_1$ on $s_2$: $\quad f\Delta y \quad \pm \quad \sigma_y , \quad d_{ML} = f\Delta y/\sigma_y$ (2)

The separation strength is thus lowered by the factor $f$, leading to a decrease of the ML effect size by the same factor and lower classification accuracy. Application of $M_2$ on $s_1$ gives the same result.

**C**. Thus far we considered models that were built from homogeneous samples (with mutual heterogeneity). We now combine the two samples $s_1$ and $s_2$ into a larger, heterogeneous sample $s_{12}$ and build a model $M_{12}$ from it. The shared features will be discriminate in both subsamples ($s_1$ and $s_2$), but the subsample-specific features are only discriminative in half of the subjects. The weights of shared features will thus be as large as in the separate models, but weights of the specific features will be half as large. More generally, if a feature has different discriminative effect sizes in different subsamples, the feature's weight is the average of the weights this feature would have in the subsample-based models. We tested this by simulations, see section F, below; and see (Dluhoš et al, in review) for tests using real MRI data. The separation strength and standard deviation of the model's output will change accordingly, with respect to the single-subsample models:

$M_{12}$ on $s_1$: $(½+f/2)\Delta y \quad \pm \quad \sqrt{(½+f/2)}\sigma_y$, $\quad d_{ML} = \sqrt{(½+f/2)}\Delta y/\sigma_y$ (3)

(Application to $s_2$ gives the same result.) The ML effect size of this two-fold heterogeneous sample is thus lowered by a factor $\sqrt{(½+f/2)}$ as compared to a homogeneous sample.

**D**. The results of the previous paragraphs on two-fold heterogeneous (sub)samples can be extended to $H$-fold (mutual) heterogeneity, with $H$=2, 3, .. (Fig. 3b). A model $M_J$ built from a combined, or joint, sample consisting of $H$ subsamples $s_1$, .., $s_H$, applied to the subjects of subsample $s_k$, ($k$=1,..,$H$) gives:

$M_J$ on $s_k$: $((1+(H-1)f)/H)\Delta y \quad \pm \sqrt{((1+(H-1)f)/H)}\sigma_y$, $d_{ML} = \sqrt{((1+(H-1)f)/H)}\Delta y/\sigma_y$ (4)

and, when such a model is tested on an independent, heterogeneous, test sample $s_T$, we find:

$M_J$ on $s_T$: $((T/H+(H-T/H)f)/H)\Delta y \ \pm \sqrt{((1+(H-1)f)/H)}\sigma_y$, $d_{ML} = ((T/H+(H-T/H)f)/\sqrt{H})/\sqrt{(1+(H-1)f)}\Delta y/\sigma_y$ (5)

where we assumed a $T$-fold overlap between the subsample-specific feature sets (see Fig. 3c) ($T$=0, 1, ..) and, as always, a shared core set of features. (For simplicity the heterogeneity of $s_T$ was assumed to be the same as that of $s_J$, i.e. $H_T = H$.)

Equations (1)–(5) can be used as a 'first order approximation' to estimate the effects of sample heterogeneity on LOOCV train and independent test accuracies. While the derivation was done for integer H and T, in practice any real value ≥0 can be substituted for these variables. This can, e.g., be of use when estimating the heterogeneity in a large sample of size $N_J$: if a typical homogeneous subsample size $N_0$ is assumed, $H= N_J/N_0$. The value of $N_0$ can be estimated from the studies in Figure 1: as long as, for growing sample size $N$, the LOOCV accuracy of the studies with highest accuracy stays the same (~90%) we can assume that the samples are homogeneous; as soon as the accuracy starts to drop, samples apparently have become heterogeneous. In fact, this sets an upper limit for $N_0$, since some "small-$N$" studies could have been able to stretch the homogeneity beyond $N_0$, by putting as much effort as possible to impose their strict inclusion criteria (which guarantee homogeneity) on as many as subjects as possible. From the figure we find $N_0 \leq 50$.

Inserting $d_{ML0}$=2.0, $f$=0, $N_0$=50, and $N$=$N_0 \times H$ in equation (4), we calculated $d_{ML}(N)$ and $\Phi(d_{ML}/2)$, the LOOCV accuracy curve in Figure 1.

For the studies in Fig. 1 that used independent train and test samples, we converted the train and test accuracies to ML effect sizes, $d_{ML}=2\times\Phi^{-1}(acc)$; inserting the ratio of the test/train effect sizes in equation (2), we calculated mutual heterogeneity factors *f*.

An approximate formula for the changes in accuracy due to (increased) heterogeneity can be obtained for accuracies in the range 70–90%. For $0.7 < \Phi < 0.9$, $acc = \Phi \approx 0.841 + 0.229 \ln(d_{ML}/2)$. Multiplication of the effect size by a factor *f* means a change in *acc* of 0.229 ln(*f*), or $\Delta acc \approx 53\%\times{}^{10}\log f$. Using the same approximation, eq. (3) becomes: $\Delta acc \approx 26\%\times{}^{10}\log(½+½f)$.

E. Derivation of the multiplication factors for $\Delta y$ and $\sigma_y$, and the relationship between *f* and the weight vectors.

Assume that discriminative features are uniformly distributed over the shared and specific subsets, i.e., the mean weight of features in the shared part equals that of the specific part(s), and can, without loss of generality, be set to 1. This means that the mean weights $w_1$ of model $M_1$ are 1 in both the shared subset with size $A_{12}$ and in the unique part with size $A_1$-$A_{12}$. We also assume that the total number of features (either discriminative or not) is $A_0$, and that thus $A_0$-$A_1$ weights of $M_1$ are zero. The squared norm of the weight vector is $||w_1||^2 = A_1$. Application of $M_1$ to $s_1$, i.e. calculating the dot product between $w_1$ and $x$ (a feature vector from sample 1), results in a separation strength of $\Delta y_1 = A_1\Delta x$, where $\Delta x$ is the mean difference of the discriminative feature values between patients and controls. Regarding the variation in the output values, $\sigma_y$, assuming noise in that is independent between the features, with mean 0 and standard deviation $\sigma_x$, the application of M1 to s1 gives rise to $\sigma_y = \sqrt{A_1}\sigma_x$. The same formulas apply to $M_2$ ($w_2$) and its application to $s_2$, but note that the sets of non-zero weights differ between $M_1$ and $M_2$ (see Fig. 3B). The inner product of the two weight vectors is $A_{12}$, and the cosine of the angle between them is $\cos(\theta) = A_{12}/A_1 = A_{12}/A$ for equally sized discriminative feature sets. Cross-sample application of $M_1$ to $s_2$ (and vice versa) gives a separation strength of $\Delta y_{1->2} = \Delta y_{2->1} = A_{12}\Delta x = fA\Delta x = \cos(\theta)\Delta y_1$. The variation in output values, $\sigma_y$, remains unchanged, as compared to the application of the models to their own sample, since it is not relevant whether a feature is discriminative or not for the noise in it to be carried over to the output value. Consequently, the ML effect size is scaled by a factor $f= \cos(\theta)$, as compared to the reference: application of a model to its own sample (using LOOCV). The angle θ is thus directly related to the heterogeneity factor *f* and we will thus call it the angle of heterogeneity. An angle of 0° reflects perfect mutual homogeneity, while an angle of 90° is found if there are no shared features: perfect mutual heterogeneity. Practically, this means that if we have two classification models acting in the same feature space, the dot product of the weight vectors directly provides us with the heterogeneity factor.

Until now, we described heterogeneity in the form of shared and unshared, i.e., specific, features. There is, however, a second form of heterogeneity, viz. features that are in contradiction between (sub)samples. This happens when there is a healthy value (or range) for a certain feature (e.g. thickness of a certain cortical region), and both smaller and larger values of this feature is associated with having the disease (thus, in some patients, included in sample 1, thickness is reduced, while in other patients, included in sample 2, thickness is increased). Linear classification models will not be able to use this feature to discriminate between patients and controls, since no single separation line can be drawn. (Transforming the feature could solve this problem, but would require a priori knowledge; invoking nonlinear kernels could also help, but

at the danger of overfitting and complicating interpretability of the model.) This kind of heterogeneity results in weights of opposite signs in the unshared feature sets. Applying model $M_1$ to sample $s_2$ (and vice versa) results in a destructive contribution of these features to the classification; assuming weights that are of the same magnitude as those in the shared feature set (1), we find: $\Delta y_{1\text{->}2} = \Delta y_{2\text{->}1} = (A_{12} - (A - A_{12}))\Delta x = (2A_{12} - A)\Delta x$. The variation in $y$ remains unchanged ($\sigma_y = \sqrt{A}\sigma_x$) and the ML effect size is thus lowered by a factor $f = (2A_{12} - A)/A$, which equals, again, cos(θ) for this case. We thus see that cos(θ) is really a measure of (different forms of) heterogeneity related to the ML effect size and thus the prediction accuracy. The angle summarizes the total heterogeneity in a single number; the precise 'shape' of the heterogeneity can be found from an element-wise comparison of the two weight vectors.

When building a model on the combined sample, the weights in the subsample-specific parts of the feature set will be the average of the weights of the single-sample models. For the non-destructive heterogeneity: $w_{12}=(w_1+0)/2 = ½$. Application of $M_{12}$ to $s_1$ (and $s_2$) gives: $\Delta y_{12\text{->}1} = \Delta y_{12\text{->}2} = (A_{12}+(A-A_{12})½)\Delta x = ½(A+A_{12})\Delta x = ½(1+\cos(\theta))A\Delta x$. The variation in $y$ becomes: $\sqrt{}(A_{12}+2(A-A_{12})(½)^2)\sigma_x = \sqrt{}(½(A+A_{12}))\sigma_x = \sqrt{}(½(1+\cos(\theta))A)\sigma_x$. The ML effect size is thus lowered by a factor $\sqrt{}(½(A+A_{12})/A) = \sqrt{}(½(1+\cos(\theta))$. One can easily verify that for the destructive type of heterogeneity the same formula holds.

Of course, in reality the heterogeneity does not manifests itself in a binary fashion (being or being not discriminative, or being exactly contradictionary), but in a continuous manner. This, however, can be equally well described by the above formulas using $f = \cos(\theta)$.

**F**. Simulations

We simulated sets of 200 features for 200 controls and 200 patients. For each simulation run, only a small fraction (5-10%) of the features were chosen to be discriminative between the groups, the other features had zero mean for both groups. The subjects were divided into 8 subsamples. For each of the subsamples, effect sizes $d_s$ were assigned to the discriminative features by sorting the absolute values of random drawings from a normal distribution with mean=0 and SD=1. This resulted in 8 heterogeneous subtypes of abnormalities. To further enhance the heterogeneity, for each of the subsamples on average 20% of the effect sizes were randomly reset to zero. For each of the subsamples, noise widths $\sigma_s$ were drawn from a normal distribution with mean=0.75 and SD=0.25. For each of the features, noise widths $\sigma_f$ were drawn form a normal distribution with mean=0.75 and SD=0.05. A total noise width $\sigma_T$ was calculated for each (subsample,feature) combination: $\sigma_T^2 = \sigma_s^2 + \sigma_f^2$. For all subjects feature values were calculated by drawing random noise from a normal distribution with mean=0 and SD= $\sigma_T$, and adding $d_s$ for discriminative features in patients. An SVM was trained to separate the patients from the controls, resulting in a model with weight vector $w$. To test whether the weights were the average of the weights of the 8 hypothetical subsample models, we also trained SVMs on these subsamples and compared the average of the resulting weights with the sample's $w$. For 140 simulations we found correlations of 0.81–0.94 between the weights.

**G**. Simple scripts to calculate machine learning effect sizes and some other measures

Please see below for a Matlab script `MLeffectsize.m` that calculates:

sensitivity = TP/(TP+FN)

specificity = TN/(TN+FP)

balanced accuracy $acc_{\text{bal}}$ = (sensitivity+specificity)/2

total accuracy $acc_{\text{tot}}$ = (TP+TN)/(TP+FN+TN+FP) = ($N_{+1}$×sensitivity+$N_{-1}$×specificity)/($N_{+1}$+$N_{-1}$)

mean output value for the '+1' and '-1' classes: $y_{+1}$ and $y_{-1}$ and their difference: $\Delta y = y_{+1} - y_{-1}$ and their standard deviations: $s_{+1}$ and $s_{-1}$ and the pooled standard deviation $s_y = \sqrt{(½s^2_{+1}+½s^2_{-1})}$

Area under the curve ($AUC$) = '$A$' ≈ '$CL$', where '$A$' is the effect size defined in Ruscio (2008), which can be approximated by '$CL$', the 'Common language effect size', defined by McGraw and Wong (1992).

Four estimates of the machine learning effect size, $d_{\text{ML}}$:

$d_{\text{ML1}} = \Delta y/s_y$

$d_{\text{ML2bal}} = 2\Phi^{-1}(acc_{\text{bal}})$

$d_{\text{ML2tot}} = 2\Phi^{-1}(acc_{\text{tot}})$

$d_{\text{MLAUC}} = \sqrt{2}\Phi^{-1}(AUC)$

$d_{\text{ML1}}$ and $d_{\text{MLAUC}}$ are calculated using the distribution of the $y$-values, whereas $d_{\text{ML2bal}}$ and $d_{\text{ML2tot}}$ use information from only one point on the ROC curve. $d_{\text{MLAUC}}$ is more robust against outliers (Ruscio (2008); McGraw and Wong (1992)). Note that accuracy should be entered in the formulas as a fraction, not as a percentage.

*AUC* can also be converted to *d*, and vice versa, with the following Excel script 'Converting effect sizes' at http://www.stat-help.com/spreadsheets.html

References :

McGraw KO, Wong SP. A common language effect size statistic. *Psychol Bull* (1992) 111:361–5. doi:10.1037/0033-2909.**111**.2.361

Ruscio J. A probability-based measure of effect size: robustness to base rates and other factors. *Psychol Methods* (2008) **13**:19–30. doi:10.1037/1082-989X.13.1.19

```
%
% MLeffectsize.m - script to calculate several effect sizes from
%                  individual prediction model [machine learning (ML)] output
%
% Reads a two-column text (ascii) input file:
% 1st column is a subject's label (-1,+1), 2nd column is the subject's predicted value
%
% The script calculates the following ML effect sizes:
%
% dML1    = dML from means and variances of y (ML output value)
% dML2bal = dML from balanced prediction accuracy
% dML2tot = dML from total prediction accuracy
% dMLAUC  = dML from area under the curve (AUC)
%
% and produces an ROC plot
%
% Hugo Schnack, Neuroimaging, Dept. Psychiatry, UMC Utrecht, 2016
%
% Uses either perfcurve() from Statistics_Toolbox - also plots ROC curve
% or fastAUC() from  http://nl.mathworks.com/matlabcentral/fileexchange/41258-faster-roc-
auc/content/install.m
% NB. In Matlab R2013a, "abs(X1-X2)" had to be replaced by "max(X1-X2,X2-X1)" in fastAUC.cpp
% other implementations of AUC() exist
%

USEfastAUC=0;   % 0 for perfcurve, or 1 for fastAUC

if( USEfastAUC == 1)
  mex fastAUC.cpp; %Compile
end

labely=load('labely.dat','-ascii');

label=labely(:,1);
y=labely(:,2);

ypos=y(label>0);
yneg=y(label<0);
Npos=size(ypos,1);
Nneg=size(yneg,1);
Nposok=size(ypos(ypos>0),1);
Nnegok=size(yneg(yneg<0),1);

sens=Nposok/Npos
spec=Nnegok/Nneg
accbal=(sens+spec)/2
acctot=(Npos*sens+Nneg*spec)/(Npos+Nneg)

mpos=mean(ypos);
mneg=mean(yneg);
spos=std(ypos);
sneg=std(yneg);
spooled=sqrt(((Npos-1)*spos*spos+(Nneg-1)*sneg*sneg)/(Npos+Nneg-2));
dy=mpos-mneg;

if( USEfastAUC == 1 )
  AUC = fastAUC(label', y', 1) % using cpp file
else
  [rocx, rocy, roct, AUC] = perfcurve(label,y,1);
  AUC
  plot(rocx,rocy);
end

dML1=dy/spooled;

dML2bal=2*norminv(accbal,0.,1.);

dML2tot=2*norminv(acctot,0.,1.);

dMLAUC=sqrt(2.)*norminv(AUC,0.,1.);

dML1__dML2balanced__dML2total__dMLAUC = [dML1 dML2bal dML2tot dMLAUC]
```
